# Suicide Risk in Older Adulthood: Differential Pathways Based on Race

**DOI:** 10.1093/geroni/igaa057.988

**Published:** 2020-12-16

**Authors:** Matthew Fullen, Mary Mize, Jihee Hong, Laura Shannonhouse, Jordan Westcott

**Affiliations:** 1 Virginia Tech, Blacksburg, Virginia, United States; 2 Georgia State University, Atlanta, Georgia, United States

## Abstract

Late-life suicide is a complex public health issue, and older adults have a higher risk threshold than the national average (Drapeau & McIntosh, 2020). Most late-life suicide research focuses on elevated risk of older white males, and less is known about risk factors among Black older adults (Joe et al., 2014). Although fewer Black older adults die by suicide than White older adults, forms of suicidality do not differ between Black and White older adults (Cohen et al., 2008). Suicide risk factors, such as psychological distress (Watkins & Johnson, 2018) and chronic pain (Bazargan et al., 2016), are prevalent among Black older adults. According to the Interpersonal Theory of Suicide (IPTS; Van Orden et al., 2016), thwarted belongingness and perceived burdensomeness inform the development of suicidal desire. These findings have been corroborated among older adult samples, though lacking racial diversity. To better understand how the IPTS functions for older adults, and probe whether suicide risk pathways operate differently depending on race, we used data from over 400 homebound older adults residing in a U.S. metropolitan area to clarify if this suicide risk pathway is similar for Black and White older adults. Race moderated the relationship between physical and psychological pain and thwarted belongingness and perceived burdensomeness, with pain among Black older adults having a greater impact on their sense of belonging and burdensomeness. Findings illuminate the need for culturally nuanced understandings of suicidality in older adulthood. The presenters will demonstrate these results and discuss implications for cross-cultural suicide prevention frameworks.

